# Neurocognitive Model of Schema-Congruent and -Incongruent Learning in Clinical Disorders: Application to Social Anxiety and Beyond

**DOI:** 10.1177/17456916221141351

**Published:** 2023-02-16

**Authors:** David A. Moscovitch, Morris Moscovitch, Signy Sheldon

**Affiliations:** 1 Department of Psychology and Centre for Mental Health Research & Treatment, University of Waterloo; 2 Rotman Research Institute and Department of Psychology, Baycrest Centre for Geriatric Care; 3 Department of Psychology, University of Toronto; 4 Department of Psychology, McGill University

**Keywords:** anxiety/stress disorders, memory, mental simulation, neuroscience, social cognition, treatment

## Abstract

Negative schemas lie at the core of many common and debilitating mental disorders. Thus, intervention scientists and clinicians have long recognized the importance of designing effective interventions that target schema change. Here, we suggest that the optimal development and administration of such interventions can benefit from a framework outlining how schema change occurs in the brain. Guided by basic neuroscientific findings, we provide a memory-based neurocognitive framework for conceptualizing how schemas emerge and change over time and how they can be modified during psychological treatment of clinical disorders. We highlight the critical roles of the hippocampus, ventromedial prefrontal cortex, amygdala, and posterior neocortex in directing schema-congruent and -incongruent learning (SCIL) in the interactive neural network that comprises the autobiographical memory system. We then use this framework, which we call the SCIL model, to derive new insights about the optimal design features of clinical interventions that aim to strengthen or weaken schema-based knowledge through the core processes of episodic mental simulation and prediction error. Finally, we examine clinical applications of the SCIL model to schema-change interventions in psychotherapy and provide cognitive-behavior therapy for social anxiety disorder as an illustrative example.

Schemas are mental representations of self, others, and the world. Derived from personal experiences (Bartlett, 1932; Neisser, 1986), schemas exert a powerful influence on the way people organize and interpret representations of past, present, and future autobiographical events. Because event representations are critical for guiding people’s thoughts, actions, and behaviors, understanding how schemas are formed and modified has significant implications for mental health and general well-being.

Negative schemas that reflect core beliefs about the self have long been identified as a central factor in the development and persistence of psychopathology as well as its treatment (Beck, 1976; Padesky, 1994; J. E. Young, 1990; J. E. Young et al., 2003). Common examples of negative self-schemas are “I am unlovable” and “I am incompetent.” Sometimes, the negative schema also encompasses the situation that interacts with the self, as in “I am socially undesirable.” When activated, these negative beliefs elicit negative emotions and sustain maladaptive patterns of behavior.

Effective cognitive-behavioral-therapy (CBT) interventions for psychopathology are thought to target and change negative schemas through the process of *new learning* acquired through personal experience (Bruijniks et al., 2019; Huppert et al., 2020; D. A. Moscovitch, Antony, & Swinson, 2009). By providing relevant contexts for patients to engage in new learning opportunities, therapy aims to help them gather evidence that challenges deeply held beliefs about self, others, and the world. Changes to maladaptive schemas during treatment are thought to promote meaningful and enduring mental-health benefits (Beck, 1976), including symptom reduction and enhanced emotional well-being, to support valued goal-directed behaviors (Rottenberg & Kashdan, 2022).

## Definition of Schema-Congruent and Schema-Incongruent Learning

We propose that the learning that occurs during effective schema-change interventions can be most clearly conceptualized as a two-part process that consists of both (a) weakening the influence of negative, maladaptive schemas (e.g., “I am undesirable to others”; “I am socially incompetent”; “Social situations are threatening”) and (b) strengthening the influence of positive, adaptive schemas (e.g., “People care about me”; “I am socially capable”; “Social situations can be warm”). Aspects of these two processes certainly overlap, both psychologically and in the brain, so they should be viewed as synergistic and complementary rather than separate or mutually exclusive. Nevertheless, as we describe below, conceptualizing the nature of these two components independently may be helpful for designing and implementing clinical interventions in ways that most effectively harness the neural organization of the brain’s autobiographical memory system to stimulate schema change.

To this end, we adopt the term “schema-incongruent” learning to reflect the therapeutic process of encoding new information that is *inconsistent* with the activated schema, with the intended goal of *weakening* the activated schema. Conversely, we introduce the term “schema-congruent” learning to reflect the therapeutic process of encoding information that is *consistent* with the activated schema, with the intended goal of *strengthening* the activated schema. Thus, when applied to treatment, engagement in schema-incongruent learning processes would be expected to weaken an activated maladaptive schema, while engagement in schema-congruent learning processes would be expected to strengthen an activated adaptive schema.

Below, we conceptualize schema-congruent and -incongruent learning through the lens of a brain-based model that emphasizes the functional interactions among the hippocampus, ventromedial prefrontal cortex (vmPFC), amygdala, and posterior neocortex during autobiographical memory encoding and retrieval. We are aware that autobiographical memory and schema formation and representation depend on larger networks whose structures interact with one another, but here we focus on these four, which constitute a process-specific assembly (Cabeza et al., 2018; Cabeza & Moscovitch, 2013). A process-specific assembly is a small team of brain regions that rapidly assemble to mediate a cognitive process in response to task demands but quickly disassemble when the process is no longer needed. We focus on this specific process-specific assembly because the encompassing four regions consistently interact with each other as part of the autobiographical memory network and have been implicated in processes that we identify as being crucial for treatment: schema reinstatement and instantiation (vmPFC^1^), episodic memory and episodic simulation (hippocampus), episodic elaboration (posterior hippocampus and posterior neocortex), and processing emotions (amygdala). On the basis of our proposed neurocognitive schema-congruent and incongruent learning (SCIL) model, we derive guiding principles for designing and administering targeted psychological interventions to facilitate schema-based learning in clinical contexts. We then illustrate how to apply the SCIL model to schema-change interventions in the context of CBT for social anxiety. Although the examples we provide pertain to CBT for social anxiety disorder (SAD), the central role of maladaptive self-schemas has been well established in other clinical disorders, including depression, posttraumatic stress disorder, and others. Thus, we assume the SCIL model would be broadly applicable to schema-based learning during treatment for a range of problems across diverse settings, therapeutic orientations, and clinical populations, and we encourage readers to extrapolate from our examples within the context of CBT for SAD to the administration of schema-change interventions for other mental disorders across therapeutic orientations.

## Autobiographical Memory and the Neural Basis of Schema-Congruent and Incongruent Learning

### Nature and function of schemas and mental simulations

Cognitive researchers define schemas as knowledge structures that reflect generalized ideas or beliefs. For example, what happens at a “typical” birthday party (event or situation schemas), what “kind” of person one is (self-schemas), or what other people are “generally” like (schemas about other people; Ghosh & Gilboa, 2014). Schemas are formed from commonalities among nonoverlapping personal episodic experiences. The relation between schemas and personal episodic experiences is bidirectional such that personal experiences are the basis for schema formation, and schemas, in turn, bias the interpretation and reconstruction of experiences by acting as a generalized framework through which the event is perceived, recollected, or imagined. Thus, accessing schemas offers the ability to recall past experiences or consider future experiences flexibly, in ways that strengthen or update schemas or change the mental representations of imagined future outcomes (Schacter et al., 2007).

The ability to recollect past autobiographical events or imagine future ones depends on episodic memory processes (or event memory processes; see Rubin & Umanath, 2015) that govern the construction of “mental simulations.” Simulations facilitate mental time travel, allowing people to relive their personal pasts and project themselves into imagined futures to help guide an individual toward pursuing valued life goals (Addis, 2020; M. Moscovitch et al., 2016; Sheldon & Levine, 2016; Sheldon et al., 2019). In contrast to schemas, mental simulations are necessarily context- or event-specific and can be derived with different levels of specificity, from “gist” to “detailed.” Whereas detailed simulations contain information about the precise perceptual details of an event, such as a birthday party (the color of the cake, its size, its location on the table, the decorations in the room), gist-based simulations contain a summary of a specific event consisting of its central elements without accompanying peripheral perceptual details. Simulations also contain appraisals about the event, including information about the arousal and valence of experienced emotions (e.g., I was very happy at that birthday party; Andrews-Hanna & Grilli, 2021; Robin & Moscovitch, 2017; Sheldon et al., 2019; Wardell et al., 2022). Thus, as we use the term, *mental simulations* are “imitative cognitive constructions of hypothetical events or reconstructions of real events” (Sanna, 2000, p. 168), which are typically imagery-based, retain a context-specific episodic signature (whether gist-like or detailed), and involve “the process of self-projection into alternate temporal, spatial, or hypothetical realities” (Waytz et al., 2015, p. 336).

### Neural basis of mental event simulations and autobiographical memory

**Fig. 1.**
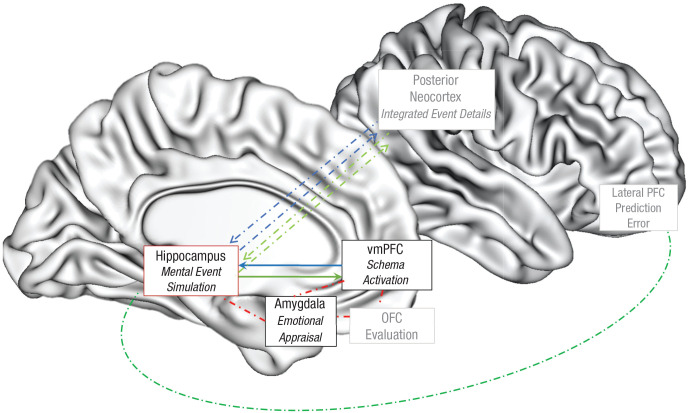
Proposed schema-congruent and -incongruent learning (SCIL) model illustrating the neurocognitive processes of mental simulation, schema processing, and schema updating. When an individual is cued to remember or simulate an event, the ventromedial prefrontal cortex (vmPFC) activates a schema that guides how the hippocampus constructs a mental simulation of this event (blue arrows). Interconnected regions (in red) between these structures provide the emotional component (amygdala) and evaluations (orbitofrontal cortex [OFC]) of the schema and event that affect the nature of schema- and event-simulation processes. An active dominant schema will drive the connected hippocampus to access and associate content in the posterior neocortex that is congruent with the schema (blue arrows). When an active schema is challenged and the posterior cortical event details included in a mental simulation are schema-incongruent, a prediction error is signaled and detected by hippocampal and associated regions, including those in the lateral prefrontal cortex, and this prediction error drives updating of the schemas represented in the vmPFC (green arrows).

As reviewed above, an autobiographical memory or event simulation is a multifaceted mental representation of an experience. These memories and simulations contain information about schemas, gist, and specific event-based details as well as emotions and appraisals of that event. The research literature suggests that each of these aspects is mediated by different structures (Fig. 1): vmPFC for schemas, anterior hippocampus for gist, posterior hippocampus and posterior neocortex for perceptual details,^2^ and amygdala (and orbitofrontal cortex) for emotions. These brain regions are functionally connected and bound together at encoding by the hippocampus to form a memory trace of the autobiographical event. However, not all aspects of a memory trace are active when retrieving or simulating an event, with this activation dependent on a number of factors, including the age of the memory, the differential rates of decay (i.e., forgetting) of different aspects of the memory, the demands of the retrieval task, the person’s goals, and the current situation and accompanying cues (Gilboa & Moscovitch, 2021; M. Moscovitch et al., 2016; Sekeres, Winocur, & Moscovitch, 2018; Sheldon & Chu, 2017; Sheldon et al., 2019; for a general view, see Gollwitzer & Moskowitz, 1996; for a related but alternative conceptualization of the functional organization of structures implicated in memory, see Ranganath & Ritchey, 2012; Reagh & Ranganath, 2018).

Most research on the neural mechanisms of autobiographical memory has highlighted the importance of the functional interactions between the vmPFC and hippocampus during mental simulation (Campbell et al., 2018; McCormick et al., 2015, 2020; Schacter et al., 2012). As central parts of a larger autobiographical neural network (Cabeza & St Jacques, 2007; Rugg & Vilberg, 2013; Svoboda et al., 2006), the vmPFC and hippocampus are consistently active in neuroimaging studies on episodic memory and imagination, including mental simulation (Schacter et al., 2012, 2017). Because the vmPFC and hippocampus play interconnected but distinct roles when a person is perceiving, retrieving, or imagining an autobiographical event (McCormick et al., 2018), damage to either one of these regions leads to profound but dissociable impairment in autobiographical memory functions, including both retrieving past life events and thinking about future ones (McCormick et al., 2018; Race et al., 2011).

For mental event simulations, the primary function of the vmPFC is to reinstate or instantiate the contributions of schemas, whereas the hippocampus functions to support the episodic memory processes used to form a contextualized and detail-rich representation of an event (Buckner & Carroll, 2007; D’Argembeau, 2013; Gilboa & Marlatte, 2017; M. Moscovitch et al., 2016; Robin & Moscovitch, 2017; van Kesteren et al., 2012). The relative contributions of the vmPFC and hippocampus vary during different stages of constructing a mental simulation. During the initial stages of such construction, when a person is cued to remember or imagine an event, the vmPFC reinstates and then instantiates an associated schema (Gilboa & Moscovitch, 2017; Giuliano et al., 2021). This schema then serves as a template for recovering the gist of an event mediated by the anterior hippocampus to construct and interpret the cued event (Bertossi et al., 2016; Dafni-Merom & Arzy, 2020; D’Argembeau, 2020; McCormick et al., 2015, 2020; Robin & Moscovitch, 2017). During a later elaboration stage, these reinstated schema and gist processes can act as top-down signals that then dictate and constrain how the hippocampus—particularly the posterior hippocampus (as noted above)—constructs and elaborates on the details of a specific episodic event via its connections to perceptual representations in posterior neocortex (Nawa & Ando, 2019). Concurrent activation and interaction between the anterior hippocampus and the closely connected amygdala (with other regions, including orbitofrontal cortex) direct event appraisals and determine the emotional tone and which informational components of a mental simulation are activated (Kim et al., 2011; Sergerie et al., 2008; Talmi, 2013).

### Neural basis of schema-congruent and -incongruent learning

By virtue of its role in processing schemas, the vmPFC plays a crucial role in monitoring the encoding and construction of memories and mental simulations to ensure that they conform to expectations consistent with the schema. When operating normally, the vmPFC’s schema and monitoring functions enable people to distinguish between schema-congruent and schema-incongruent experiences and memories, whether externally driven or internally generated, and adjust their behavior accordingly.

As noted above, there are bidirectional connections between the vmPFC and hippocampus that are critical for mental simulation, and these pathways activate important subcortical areas relevant for the appraisal aspect of the simulation, including the amygdala that supports emotional appraisals (Bechara & Damasio, 2005; Rangel et al., 2008). Critically, the nature of the interaction between the vmPFC and the hippocampus during mental simulation—and the activity of these intervening regions—is thought to depend on the content included in the simulation (Greve et al., 2019; Ritchey & Cooper, 2020; Sheldon et al., 2019; van Kesteren et al., 2012; Wing et al., 2022).

A prominent factor that determines the nature of this interaction is whether a simulated event emphasizes content that is congruent or incongruent with an activated schema (Bonasia et al., 2018). During the construction of schema-congruent mental simulations by the hippocampus, the vmPFC activates detailed content that is relevant to the schema while also inhibiting content that is schema-irrelevant (Bonasia et al., 2018; van Kesteren et al., 2012; van Kesteren & Meeter, 2020). Through this process, the hippocampus binds together only details of a remembered or imagined experience that fit with the schema, thus forming a schema-congruent mental simulation of an event (Addis, 2020; Schacter, Addis, & Buckner, 2007). Through consolidation, these schema-congruent simulations are rapidly assimilated into the schema so that—except for the most salient memories—they lose their detailed representations and retain only their gist or schema-congruent information (Audrain & McAndrews, 2022; Tse et al., 2011).

Schema-incongruent learning occurs when hippocampal processes construct a mental simulation that violates or challenges the expectation generated by the vmPFC-supported schema, triggering a prediction error. The prediction error is then detected by the vmPFC and other structures involved in monitoring, such as the lateral prefrontal cortex ([PFC]; Botvinick et al., 2011; Gilboa & Marlatte, 2017; Joiner et al., 2017; Moscovitch & Winocur, 2002), and drives the hippocampus toward encoding—and generating—specific contextual details of an event, including those that are not congruent with a schema (Gruber & Ranganath, 2019; Greve et al., 2019; Nyberg, McIntosh, & Tulving, 1997; Sinclair & Barense, 2019; Strange et al., 2005). Mnemonic prediction errors are known to engage updating mechanisms of associated schemas, likely for the purpose of improving predictions of these schemas (Bein, Duncan, & Davachi, 2021; Henson & Gagnepain, 2010). Experiences that promote the formation of detailed, schema-incongruent hippocampal memories will either force an activated schema to change or lead to the formation of new schemas to accommodate these incongruent events (Brod et al., 2013; Kumaran, 2013; Levy & Schiller, 2021; Ritcher et al., 2019; Schultz et al., 1997; Strange et al., 2005; Sutton & Barto, 1981).

**Fig. 2.**
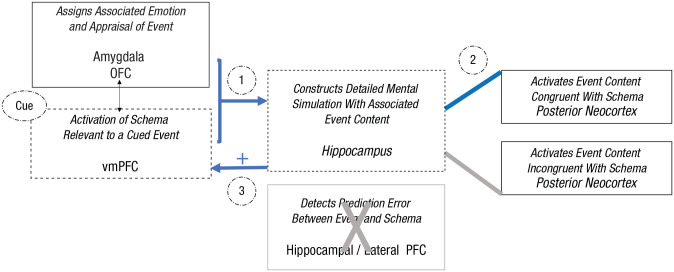
Illustration of schema-congruent learning processes within the proposed neurocognitive schema-congruent and -incongruent learning (SCIL) model, focusing on the roles of the hippocampus and ventromedial prefrontal cortex (vmPFC). When a cue triggers the recovery or imagination of an event, (1) an associated schema will be activated by the vmPFC to direct the construction of the detailed episodic mental simulation of the event by the hippocampus (blue arrows). (2) Driven by the vmPFC schema, the hippocampus will associate event details processed in the posterior neocortex that are congruent with the schema. (3) This episodic mental simulation constructed with only schema-congruent event details will confirm the predictions of the schema and thus strengthen the associated representation.

As shown in Figure 2, during schema-congruent learning, when an episodic event is simulated primarily with content that meets the expectations of an activated schema, the event representations in the vmPFC and hippocampus will reinforce one another, at least during encoding and at short delays (Bonasia et al., 2018; Dusek & Eichenbaum, 1997; Schlichting et al., 2015; Zeithamova et al., 2012; Zeithamova & Preston, 2010). At longer delays, detailed event-specific information will decay or be forgotten more quickly than gist and schema information (Sekeres et al., 2016). At longer delays, even gist may be lost, leaving only the schema-reinforced and schema-congruent information; in other words, the congruent episodic memory becomes assimilated into the schema such that at delayed retrieval, the ability to recall detailed information about a congruent memory is quickly forgotten as the gist of that memory is incorporated into the matching schema.

**Fig. 3.**
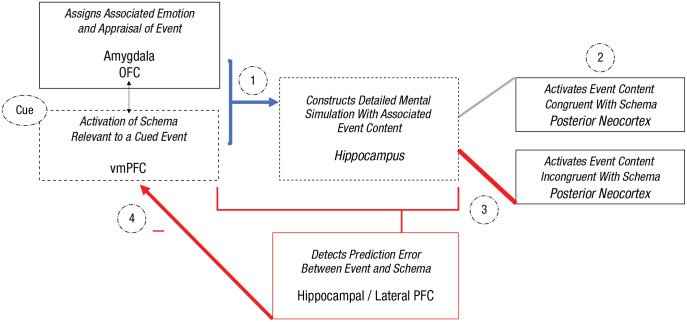
Illustration of schema-incongruent learning processes within the proposed neurocognitive schema-congruent and -incongruent learning (SCIL) model, focusing on the roles of the hippocampus and ventromedial prefrontal cortex (vmPFC). When a cue triggers the recovery or imagination of an event, (1) an associated schema will be activated by the vmPFC to direct the construction of the detailed episodic mental simulation of the event, which is supported by the hippocampus (blue arrow). (2) Driven by the vmPFC schema, the hippocampus will access and associate event details processed in the posterior neocortex that are congruent with the schema but also incongruent schema details. (3) When a mental simulation of an event is constructed with salient schema-incongruent details that violate schema-congruent expectancies, a prediction error will be detected by the hippocampus and other brain regions (e.g., lateral prefrontal cortex). (4) The prediction error will lead to updating or weakening of the activated schema.

In contrast, as show in Figure 3, schema-incongruent learning occurs when there is sufficient conflict between the schema’s expectations and either the experience of an event or a constructed mental simulation to trigger a prediction error signal in the brain, which stimulates hippocampus-dependent mechanisms, working with other regions such as the lateral PFC and amygdala (Dixon & Dweck, 2021) to modify the schema with the new information (Lane & Nadel, 2020; Sinclair & Barense, 2019). A prediction error signal can emerge only if a mental simulation—the hippocampal construction of a contextualized event—challenges the appraisal and knowledge represented within the schema (Schultz et al., 1997). Thus, to induce schema-incongruent learning for self or event schemas, the hippocampus must be active in concert with the vmPFC as conflicting details are added to the constructed event. Thus, this pathway offers an opportunity for people to update the schema by assigning a new meaning to the event and/or associating a new emotional response to it, thereby enabling schema accommodation.

As described above, the connections between hippocampus and vmPFC also include mediating pathways with the amygdala and orbito-frontal cortex that support emotional evaluation and regulation of autobiographical memory constructions and simulations. Of relevance to clinical disorders, the amygdala may play a key role in schema-congruent and -incongruent learning. Mounting evidence suggests that the amygdala specializes in salience detection of emotional stimuli across the valence spectrum (i.e., both threat and reward stimuli that are relevant for evolutionary fitness); thus, the amygdala tends to be activated in response to both negative and positive autobiographical constructions (Dixon & Dweck, 2021; Pine et al., 2021). Indeed, recent research has shown that projections to the amygdala from hippocampus and vmPFC can produce either excitatory or inhibitory effects, depending on whether activation occurs within safe or threatening contexts (Pine et al., 2021; Steward et al., 2021). Thus, the amygdala may be active not only during the detection of salient, schema-congruent, anxiety-related or threat-based emotional stimuli, as suggested by past research on psychopathology, but also when prediction error and “value updating” occurs as a result of schema-incongruent learning while experiencing or simulating an event (K. D. Young et al., 2018; Zhang et al., 2020).

Emotional arousal experienced when an autobiographical event is encoded or mental simulation is formed, which is supported by the amygdala, boosts consolidation of the underlying memory trace (Holland & Kensinger, 2010) and may also enable emotionally salient features of the event to be processed more efficiently and preferentially retrieved from memory (Talmi, 2013; Talmi et al., 2019). Thus, repeated mental replay of negative emotional experiences may engender the formation of multiple, related memory traces by the hippocampus that populate the autobiographical memory system, increasing the likelihood that they are preferentially retrieved in the future (Nadel & Moscovitch, 1997; Winocur & Moscovitch, 2011). The frequent simulation of these memory details in interaction with negative emotions and appraisals may embellish and even distort the recollected experience to conform with the schema, ultimately hindering individuals’ ability over time to distinguish between real memory details and schema-congruent imagined details that feel real but may have never actually occurred (Brainerd & Reyna, 2005; Hertl et al., 2008; Loftus & Pickrell, 1995; Miller & Gazzaniga, 1998; see also M. Moscovitch, 2008).

### Summary

We propose that both schema-congruent and -incongruent learning emerge from the interplay between the hippocampus and vmPFC and their connections with amygdala and posterior neocortex. Research evidence suggests that the relative levels of vmPFC top-down influence and hippocampal bottom-up activation when constructing a mental event simulation are determined by the amount of overlap between the represented event and the expectations of the schema, which governs the initiation of either schema-congruent or -incongruent learning (Gilboa & Marlatte, 2017; Kumaran, 2013; van Kesteren et al., 2012). As described in more detail below, new learning is then organized and integrated into long-term memory over time through repeated rehearsal. Active strategies can also be applied to boost memory consolidation, with clear implications for intervention (see below).

## Applying the Neural Principles of Schema Change to Optimize CBT Interventions

### Integrating neural and clinical models of schema-based learning

Contemporary models outlining the mechanisms of schema change and symptom reduction during exposure-based CBT for emotional disorders—including emotional-processing theory (Foa et al., 2006; Foa & Kozak, 1986), competition-retrieval theory (Brewin, 2006), inhibitory-learning theory (Craske et al., 2008, 2014), and reconsolidation theory (Ecker & Bridges, 2020; Elsey et al., 2018)—all focus on the critical role of new learning (Huppert et al., 2020; D. A. Moscovitch, Antony, & Swinson, 2009). However, whether clinical interventions work by modifying schemas or by altering their strength or accessibility is debated by proponents of these theories. For example, according to retrieval-competition and inhibitory-learning theories, successful treatment works by enabling patients to learn new, more positive mental self-representations that compete with the original negative representations for subsequent retrieval within relevant contexts. However, reconsolidation theory and versions of emotional-processing theory propose that new learning during effective treatment enables the direct modification of negative mental representations (see Huppert et al., 2020; Lane & Nadel, 2020; Phelps & Hofmann, 2019).

Applying a neurocognitive model to conceptualize how new learning occurs during therapy might help to shed new light on the mechanisms involved and help to resolve these debates. We propose an integrative account of schema change that emphasizes key roles for both retrieval competition *and* direct modification of mental representations in facilitating schema malleability within the autobiographical memory system. To this end, schema-incongruent learning during treatment would be expected to modify elements of the original maladaptive schema directly via bottom-up, hippocampus-driven mental simulations of episodic experiences that violate schema-derived expectancies and generate prediction error. During schema-incongruent learning, new information is consolidated into the memory representation, resulting in schema modification within the vmPFC (Ecker & Bridges, 2020; Elsey et al., 2018). Simultaneously, however, these same hippocampal mental simulations that are incongruent with the maladaptive schema function would also be expected to generate and strengthen new, more adaptive schemas, and these alternative schemas would subsequently compete with the original schemas for retrieval. New schemas are fragile and must be reinforced within the vmPFC through repeated and detailed input of schema-congruent episodic mental simulations, thereby reducing dependency on the original schema during subsequent event constructions.

### Practical application of the neural model to clinical interventions: a three-phase process

On the basis of the literature reviewed above, we propose that effective schema-change interventions during psychotherapy can be conceptualized as a three-phase process: (a) preparation, which is identifying and activating target schemas within the therapeutic context; (b) new learning, which is weakening maladaptive schemas and strengthening adaptive schemas through repeated administration of episodic memory constructions and simulations; and (c) rehearsal, which is deepening learning and retention of encoded intervention material by facilitating consolidation processes.

#### Preparation

Identifying target schemas for individual patients requires an accurate a priori collaborative case conceptualization (Kuyken, Padesky, & Dudley, 2009). In patients with emotional disorders, the maladaptive schema and supporting personal experiences that patients can recall from their lives tend to be frequently simulated and mentally replayed, whereas retrieval of the adaptive schema and associated experiences is rarer and less familiar (Padesky, 1994; for a recent example, see Williams et al., 2022). For example, to access maladaptive self-schemas, clinicians can guide patients to use the downward-arrow technique (e.g., “If this [surface level anxious thought] is true, what does that mean about you?”; see Reimer & Moscovitch, 2015). Identifying adaptive self-schemas can be more difficult for patients with emotional disorders and other forms of psychopathology in which access to negative self-schemas predominates; thus, to generate adaptive self-schemas, clinicians can guide patients to construct a detailed vision of how they “would like” to be, encouraging them to identify the thoughts, feelings, and behaviors that support the vision (Padesky, 1994, p. 269).

#### New learning

After relevant schemas have been identified and activated within the therapeutic context, schema-change interventions, consisting at their core of hippocampus-driven mental simulations of real and/or imagined episodic events, can be applied. To weaken (old) maladaptive schemas while simultaneously strengthening (new) adaptive schemas, episodic memory constructions and simulations must carry a strong hippocampal signal. Thus, episodic simulations should be imagery-based, detailed, and context-specific. Simulations with these properties will be most likely to engage the unique encoding preferences of the hippocampus and, in turn, activate the critical schema-congruent and -incongruent learning pathways reviewed above. Studies on memory reconsolidation suggest that modification of old memories is most effective if schema-incongruent learning is applied shortly after the old maladaptive memory has been retrieved when it is in a labile state and most amenable to modification (see Elsey et al., 2018; Phelps & Hofmann, 2019).

#### Rehearsal

We propose that the episodic memory constructions and simulations introduced in the second phase must be rehearsed continually through intentional, detailed mental replay to deepen new learning and promote memory consolidation. Each rehearsal trial should begin with the reactivation of the target schemas, followed by a detailed episodic simulation that reinforces the strong hippocampal encoding properties from Phase 2. During this third phase, patients should be instructed to rehearse in a spaced interval schedule because studies have shown that spaced learning with repeated long intertrial intervals improves memory formation compared with massed training with no intervals (Smolen et al., 2016). Patients should also be encouraged to schedule discrete rest or sleep periods after simulation rehearsal to promote deeper memory consolidation and the integration of episodic memories into schemas (Azza et al., 2022; Stickgold, 2005).

During the rehearsal phase, patients might benefit from either reappraising (Gross & John, 2003) or intentionally suppressing (Stramaccia et al., 2021) the (old) memories that are associated with (old) maladaptive schemas when they come to mind while redirecting their attention to the (new) memories that are associated with (new) adaptive schemas. Studies have shown that the capacity to exert adaptive control over unwanted memories may be disrupted in clinical disorders and that recovering this capacity may be an important part of effective treatment that enables more efficient down-regulation of PFC on hippocampus (Anderson et al., 2004; Anderson & Levy, 2009; Hermann et al., 2017, 2021; Joormann et al., 2005; Mary et al., 2020; Paz-Alonso et al., 2009; Sokołowski et al., 2022). However, CBT interventions tend to promote reappraisal rather than suppression because unsuccessful attempts to suppress memories may inadvertently lead to their retrieval and further strengthen the associated schema.

### Summary

From the perspective of therapeutic goals, we propose that clinicians can design and administer effective schema-change interventions by collaborating with patients first, to identify and activate maladaptive schemas and associated episodic memories as well as their adaptive counterparts and, second, to use repeated episodic mental simulation to stimulate bottom-up hippocampal processing to shift the relative balance in the strength and accessibility of the two schema representations in the vmPFC over time. Intentional rehearsal of memory simulations supporting the adaptive schemas, paired with scheduled rest and sleep periods (Diekelmann & Born, 2010; Dudai et al., 2015) and voluntary reappraisal or suppression of old memories supporting the maladaptive schemas, is encouraged to amplify the intervention effects. In any relevant context during or between treatment sessions, both the maladaptive and adaptive schemas may be active, with a key point being the nature of their relative strength in affecting new event constructions. Through the administration of repeated trials of schema-congruent and -incongruent learning during and between therapy sessions, clinicians can aim to help their patients shift the relative strength and accessibility of their adaptive versus maladaptive schemas over time.^3^

## Applications of Neural Model to Schema-Change Interventions for Social Anxiety

Given the established links between episodic memories and schemas, it is no surprise that autobiographical event encoding and retrieval have been theorized to play a prominent role in the maintenance of symptoms in clinical disorders that are characterized by negative self-perception (Cohen & Kahana, 2022). In this section, our objective is to illustrate how the tenets and predictions of our neurocognitive SCIL model can be applied clinically to one such disorder—SAD—a common and impairing problem with a lifetime prevalence rate of approximately 12% that is characterized by marked anxiety about social situations in which negative evaluation might occur (Aderka et al., 2012; American Psychiatric Association, 2013; Kessler et al., 2012).^4^ We selected SAD as an illustrative example for the purpose of this article, but, as noted above, we believe the SCIL model is broadly applicable to a range of clinical problems and assume that clinicians will apply the ideas and principles outlined here using the exemplar of SAD to schema-change interventions administered in practice for other mental disorders with diverse clinical populations.

### Negative self-perception, self-imagery, and autobiographical memory in SAD

According to cognitive models of SAD, negative self-schemas play a central role in the development and maintenance of social-anxiety symptoms (e.g., Clark & Wells, 1995; Hofmann, 2007; D. A. Moscovitch, 2009; Rapee & Heimberg, 1997). Individuals with SAD tend to view themselves as being socially undesirable and are fearful of revealing perceived self-flaws to evaluative others within social contexts (D. A. Moscovitch et al., 2009, D. A. Moscovitch, Waechter, Bielak, Rowa, et al., 2015; D. A. Moscovitch, Rowa, Paulitzki, Antony, et al., 2015; D. A. Moscovitch & Huyder, 2011). Studies have shown that what distinguishes people with SAD from control participants is not whether they can recall having endured negative social experiences (indeed, everyone has had such experiences) but the level of detail with which such memories and associated imagery-based mental simulations are retrieved and reconstructed, the personal meaning and significance they tend to carry, and the emotional and behavioral effects of bringing them to mind (D. A. Moscovitch, 2016). Specifically, people with SAD tend to retrieve more negative details of their socially painful experiences than control participants without SAD, experience greater distress when such memories are retrieved, and appraise the meaning of these past experiences in more negative ways that maintain and reinforce negative self-perception (D. A. Moscovitch et al., 2018; Reimer & Moscovitch, 2015).

Research points to the critical role of imagery-based mental simulation in mediating the link between social anxiety and the negative consequences of autobiographical memory retrieval and rehearsal (see Morgan, 2010). For people with SAD, schema-congruent meanings derived from painful or humiliating past social experiences are encapsulated within intrusive mental images, and these images are mentally replayed before, during, and after social encounters (Chiupka et al., 2012; Cody & Teachman, 2010; Gavric et al., 2017; Hackmann et al., 2000). Continual replay of schema-congruent mental simulations, in turn, strengthens the use of such schemas as a lens through which to process and make sense of social events that are recalled, including both past and future social events (Çili & Stopa, 2015; Romano, Moscovitch, Ma, & Moscovitch, 2020), thereby strengthening schema-congruent learning. Thus, personal experiences that are consistent with negative views of self may, over time, come to be appraised by individuals with SAD as “self-defining” memories—affectively intense, repetitive, and vivid exemplars of self that inform one’s sense of identity and serve as proscriptive scripts for goal-directed action (Conway, 2005, 2009; Conway & Pleydell-Pearce, 2000; Krans et al., 2014; Singer & Salovey, 1993; Sutherland & Bryant, 2005).

### Schema-congruent and -incongruent learning biases in SAD: implications for treatment

Increasing evidence has accumulated in support of the claim that for adults with SAD, it is more difficult to learn “I am liked” than “I am disliked,” whereas the opposite is true for nonanxious adults (e.g., Button et al., 2012; Sharot & Garrett, 2016). Multiple studies have shown that people with low self-esteem or high levels of trait social anxiety, including people with SAD, fail to update their negative self-schemas in response to unambiguously positive self-relevant feedback (Beltzer et al., 2019; Everaert et al., 2018; Glazier & Alden, 2019; Hopkins et al., 2021; Koban et al., 2017; Will et al., 2020).

These findings have prompted researchers to speculate that failure to incorporate positive self-relevant social feedback in SAD is related to the suppression of neural circuitry that is responsible for directing adaptive self-schema-based learning and memory (e.g., Piray et al., 2019; Voegler et al., 2019). Difficulties in self-referential processing in SAD have been linked to individual differences in the activation of medial PFC (e.g., Blair et al., 2010, 2011), amygdala (Blair et al., 2016; Burklund et al., 2017), and amygdala-prefrontal connectivity (Cremers & Roelofs, 2016); some studies have also implicated a key role for the hippocampus in directing memory-based appraisals of specific social experiences (FeldmanHall et al., 2021; Goldin et al., 2013).

**Fig. 4.**
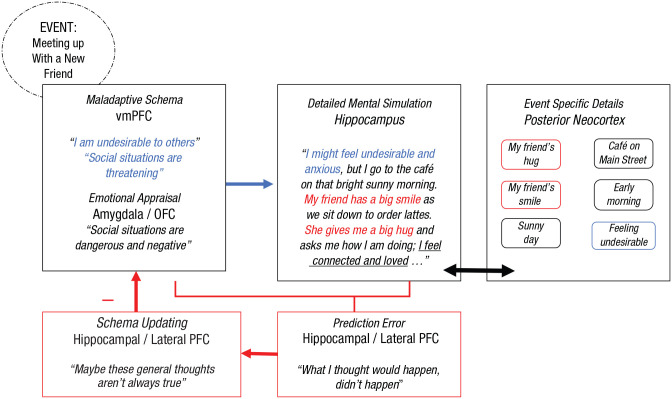
Therapeutic application of the neurocognitive schema-congruent and incongruent learning (SCIL) model to facilitate schema-incongruent learning in social anxiety disorder for the purpose of weakening the influence of negative or maladaptive schemas. In this example, a negative or maladaptive schema is active. Elements of the mental simulation that are schema-incongruent are highlighted in red, and schema-congruent elements are highlighted in blue.

**Fig. 5.**
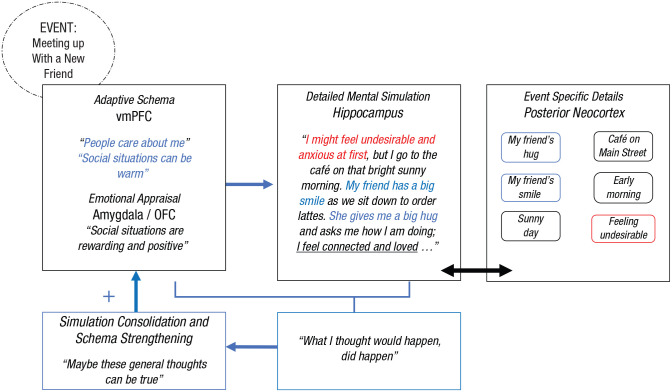
Therapeutic application of the neurocognitive schema-congruent and incongruent learning (SCIL) model to facilitate schema-congruent learning in social anxiety disorder for the purpose of strengthening the influence of positive or adaptive schemas. In this example, a positive or adaptive schema is active. Elements of the mental simulation that are schema-incongruent are highlighted in red, and schema-congruent elements are highlighted in blue.

The fact that negative self-schemas in SAD are highly resistant to updating presents a critical challenge for designing and delivering effective clinical interventions. Such resistance to self-updating may be a key reason that “gold-standard” CBT protocols for SAD are not more successful; more than 50% of patients who receive them fail to achieve high end-state functioning by the end of therapy (Rapee et al., 2009). As shown in Figures 4 and 5, optimal self-schema-updating interventions for SAD could be strategically implemented using our neurocognitive SCIL model for the purpose of both weakening negative or maladaptive schemas and strengthening more positive or adaptive ones (see Thurston et al., 2017) to change how patients view themselves and their social currency in the eyes of others across social contexts.^5^

Given these premises, what are the key insights and practical recommendations derived from the SCIL model for implementing effective interventions for SAD? How can clinicians administer interventions in ways that leverage their knowledge of the organization and functionality of the autobiographical memory system to facilitate enduring schema change? In neurocognitive terms, we argue that this core therapeutic goal can be most effectively achieved using interventions that engage bottom-up hippocampal-vmPFC pathways. Specifically, we propose that hippocampally driven, schema-based learning can be optimally engaged by interventions that (a) consist of detailed imagery-based mental simulations that are administered during schema activation, (b) contain meaning and content that violate maladaptive expectancies and reinforce adaptive ones, and (c) are processed and practiced in ways that strengthen memory consolidation. Below, we unpack each of these components in relation to the treatment of SAD.

### Clinical and behavioral evidence supporting the use of episodic mental simulation in the treatment of SAD

Research suggests that people can be trained to use mental simulation effectively to improve their coping and self-regulation in the face of stress (Hallford et al., 2020; Taylor et al., 1998). Mental simulation facilitates problem solving because it enables people to prepare for future events, interpret previously experienced events, and establish links between thought and action (Taylor & Schneider, 1989). However, stress and anxiety may block access to helpful forms of mental simulation (Brown et al., 2020; Riskind & Calvete, 2020), instead fueling a ruminative verbal worry process that inhibits adaptive problem solving in people with anxiety difficulties, including people with SAD (Borkovec & Inz, 1990; Romano et al., 2019; Stöber, 2000; Stokes & Hirsch, 2010).

Training in adaptive mental simulation improves problem solving because visualization of episodic simulations containing rich perceptual details tends to activate the hippocampus and reduce the tendency to engage in schema-congruent or generalized mental simulation (Madore & Schacter, 2014; Sheldon et al., 2011; Taylor & Schneider, 1989). Past studies in nonanxious participants have found that *process simulation*—visualizing the step-by-step process that would result in achieving a desired goal—may be particularly effective and has been shown to facilitate desired emotional and behavioral outcomes across both social and nonsocial contexts (Pham & Taylor, 1999; Sheldon et al., 2011).

Earlier, we noted that mental simulations can be either gist-based or detailed. Experimental studies have shown that detailed imagery-based simulations can serve as an “emotional amplifier” that enhances emotional-information processing (Holmes & Matthews, 2010). Recent clinical studies by McEvoy and colleagues (2014, 2015, 2018, 2022) demonstrated that detailed imagery-based simulation training can also be incorporated effectively into CBT for SAD and that imagery-based enhancements of traditional verbal-linguistic CBT interventions may help to improve treatment outcomes. Likewise, analogue-clinical studies of future-focused, detailed, episodic-simulation training in nonclinical and dysphoric samples have shown that training people to generate specific detailed scenes of imagined positive future events can help improve participants’ perception of control over future events as well as their feelings of anticipatory pleasure (Boland et al., 2018; Hallford et al., 2020).

We propose that imagery-based mental simulation is effective, at least in part, because it succeeds in activating the hippocampus (and, in turn, the vmPFC) in ways that are consistent with the processes required to promote effective updating (Schacter et al., 2007, 2012; Sheldon et al, 2011; Sheldon & Moscovitch, 2012). As reviewed above, the hippocampus is specialized precisely for constructing mental simulations that are rich in episodic detail and amenable to mental time travel (Addis, 2020). Thus, we recommend intentionally incorporating imagery-based simulation training into CBT to facilitate schema-incongruent learning. Such simulations could include retrieving specific memories of actual experiences and/or envisioning imagined constructions of hypothetical experiences because any detailed mental simulation should similarly activate the hippocampus regardless of whether it is real or imagined or past-focused or future-focused (Benoit et al., 2016; Reddan et al., 2018). As described, patients with SAD may frequently retrieve negative images of anticipated and/or past anxiety-provoking experiences (e.g., “My face turned/will turn red”; “Nothing was/will be coming out of my mouth”; “People were/will be looking alarmed or disgusted”; see Chiupka et al., 2012) that are consistent with a maladaptive schema (e.g., “I am socially undesirable”). Likewise, effective schema-change simulations must engage patients to mentally envision details from actual or hypothetical positive experiences (e.g., “She smiled at me and gave me a hug”; “We laughed and had fun together at that party”; “He made an effort to reach out to support me when I was going through a hard time”; “The teacher said I articulated my ideas clearly during that presentation”) that would serve to weaken the maladaptive schema (e.g., “I am socially undesirable”) and strengthen adaptive alternative ones (e.g., “People care about me”; “Social situations can be warm”).

To ensure that the hippocampus is optimally activated, mental simulations should include meaningful situation-specific detail rather than general or abstract concepts about an event. For example, when patients use cognitive-restructuring exercises to “examine the evidence” and challenge their negative thinking, they should incorporate mental imagery and detailed mental simulations that engage the hippocampus rather than merely articulating a verbal, schema-based conclusion (e.g., “I’m not as awkward as I thought”). To illustrate this principle more clearly, imagine patients who arrive to CBT sessions with a newly completed thought record in which they identified and challenged a negative schema-congruent thought (see Greenberger & Padesky, 2016). According to our model, an effective process for conducting the homework review may involve, first, helping the patients use the downward-arrow technique to identify a maladaptive schema that is tied to the anxiety-provoking situation and then guiding the patients to challenge the content and meaning of the activated maladaptive schema not only verbally and in general terms but also with a detailed visual simulation. This simulation might be facilitated by asking them to close their eyes and mentally relive the specific anxiety-provoking situation from their past week while incorporating the evidence against the schema within their detailed visualization. The evidence against within the simulation should then be explicitly linked to an adaptive schema and rehearsed repeatedly for homework. Finally, the patients could be guided to imagine a future hypothetical scenario in which they mentally simulate and visualize themselves successfully navigating a social situation in a manner that is consistent with the adaptive schema.

### Optimizing the effectiveness of mental-simulation-based intervention procedures for SAD

Various imagery-based mental-simulation procedures have already been shown to be effective and even essential in the treatment of SAD, perhaps in part because they are likely to engage the hippocampus and effectively stimulate schema-based learning. For example, video feedback activates negative schemas during an initial video-recorded social performance and then guides patients to use mental simulation to envision their negative mental image in detail and compare the characteristics of that image with those of an updated, more realistic image drawn from the video evidence of their performance (e.g., Harvey, Clark, Ehlers, & Rapee, 2000). In video feedback, there is an explicit emphasis on prediction error (Orr & Moscovitch, 2010); patients are led to first simulate what they will see in the video recording and then shown the recording and guided from an observer’s perspective to notice how each specific prediction in turn is violated by the video evidence, which they are encouraged to watch and simulate in their imagination repeatedly for homework—exercises that can be used both to weaken maladaptive self-schemas and strengthen adaptive alternative ones.

In addition, behavioral experiments force patients to construct new mental representations of anxiety-inducing experiences (Bennett-Levy et al., 2000). Although behavioral experiments are considered a core, essential element of most CBT protocols, mental-simulation rehearsal is not routinely assigned for homework as an integral part of treatment following successful behavioral experiments. On the basis of our model, we propose that clinicians should instruct patients to later simulate and rehearse the episodic memories of behavioral experiments to facilitate continued schema-incongruent learning designed to weaken existing negative beliefs about how they appeared to others (e.g., “I am unappealing to others”) and schema-congruent learning designed to strengthen fragile positive beliefs about the self (e.g., “Others accept me for who I am”).

Another imagery-based intervention procedure that has been shown to be effective in treating SAD is imagery rescripting (Knutsson et al., 2020; Lee & Kwon, 2013; Nilsson et al., 2012; Norton & Abbott, 2016; Reimer & Moscovitch, 2015; Romano et al., 2021; Romano, Moscovitch, et al., 2020; Wild & Clark, 2011). In imagery rescripting, patients are instructed to retrieve and simulate a negative self-defining memory in detail and then modify its characteristics in their imagination to help their younger self within the memory fulfill unmet emotional and interpersonal needs (in ways that are aligned with an adaptive self-schema). Even a single session of imagery rescripting has been shown to stimulate changes in the content and meaning of the episodic constructions and lead to greater schema change than control conditions, as reflected in reported core beliefs about the self (Reimer & Moscovitch, 2015; Romano, Moscovitch, et al., 2020).

As with behavioral experiments, clinicians administering imagery rescripting have not routinely incorporated intentional rehearsal into the postrescripting homework assignments that patients are instructed to complete in the week following interventions, although we propose that doing so could enhance treatment benefits in the manner detailed above. Indeed, basic experimental studies have shown that schema updating is strengthened in memory in accordance with the time dedicated to the consolidation of schema-inconsistent information (Korkki et al., 2021; Richter, 2020; Richter et al., 2019). Thus, after the acute administration of simulation-based interventions, clinicians should work with patients to deepen their processing of schema-incongruent information through intentional within- and between-sessions practice designed to enhance memory consolidation of new material. Analogue-clinical studies have shown that relatively simple depth-of-processing manipulations can be administered to enhance patients’ attention to aspects of schema incongruence in the aftermath of imagery-based interventions for social anxiety such as video feedback (Orr & Moscovitch, 2010) and after the intentional retrieval of positive memories (Moscovitch, White, & Hudd, submitted).

Finally, we propose that memory consolidation and learning outcomes may also be boosted during spaced retrieval trials by inducing sleep via intentional napping after encoding. Although research has been limited, select studies suggest that pairing sleep with exposure exercises can enhance new learning, and has the potential to improve the effects of CBT for SAD (Pace-Schott et al., 2018; Zalta et al., 2013). In addition to simulating and rehearsing positive memories to bolster adaptive schemas, it may be helpful simultaneously to engage in intentional reappraisal or suppression of the negative memories associated with maladaptive schemas in SAD during the rehearsal phase of treatment. Indeed, cognitive reappraisal has been established as a key mechanism underlying symptom changes during CBT for SAD (Goldin et al., 2012; D. A. Moscovitch et al., 2012). Although prior studies have linked memory suppression to adaptive forgetting of unwanted memories in healthy individuals (Stramaccia et al., 2021), few studies have investigated the potential benefits of suppressing negative memories as a strategy for improving symptoms of social anxiety (see Cougle et al., 2005; Magee & Zinbarg, 2007). More research is needed to determine whether unsuccessful attempts by individuals with SAD to suppress negative memories may backfire by inadvertently cuing their retrieval, thus strengthening rather than weakening the associated maladaptive schema.

### Summary

We recommend that clinicians ought to view mental simulation as an essential part of the therapeutic process that patients with SAD should be encouraged to practice repeatedly to support schema-based learning. Intervention techniques that incorporate imagery-based mental simulation have been shown to be effective in treating SAD, including video feedback and imagery rescripting. Our SCIL model supports the use of simulation-based interventions because they would be expected to engage the autobiographical memory system in ways that optimally activate schemas, induce schema-based learning, and bolster memory consolidation through repeated rehearsal. Our model would predict that the effects of traditional verbal-linguistic CBT interventions for SAD, such as cognitive restructuring, could be enhanced with the intentional incorporation of detailed mental simulation.

Ultimately, the cumulative benefits of simulation-based self-updating in SAD ought to stimulate the pathways from the vmPFC to downstream neural-reward centers elsewhere in the brain, outside the autobiographical memory system, which will enable socially anxious patients to begin adopting and benefiting from approach-oriented social-behavioral goals (Hudd & Moscovitch, 2020; Richey et al., 2014). Following successful schema updating, individuals with SAD should begin to engage in more frequent social-approach behaviors while also relinquishing avoidance-based self-regulatory strategies that block their ability to derive pleasure from social relationships and occupy valuable attentional resources that interfere with adaptive social problem solving and emotion regulation (see Alden et al., 2018; Barber, Michaelis, & Moscovitch, 2021; Gilboa-Schechtman et al., 2014; Kashdan et al., 2011; Moscovitch, Rowa, et al., 2013; Plasencia et al., 2016).

## Conclusion

Guided by the neuroscientific literature on episodic memory and its role in self-schema development and updating, we presented a neurocognitive SCIL model that highlights the critical roles of the hippocampus, vmPFC, amygdala, and posterior neocortex as the primary hubs of an interactive neural network that directs schema-congruent and -incongruent learning within the autobiographical memory system. After describing the SCIL model, we used it to derive new insights and predictions about the optimal design features of clinical interventions for strengthening or weakening schema-based knowledge and promoting memory updating through the core processes of episodic mental simulation and prediction error. We emphasized a three-part process to facilitate schema updating during treatment consisting of a preparation phase, a new learning phase, and a rehearsal phase. We then proposed clinical methods for generating schema change most effectively during treatment through the application of mental-simulation-based intervention strategies that promote and consolidate new learning by capitalizing on the unique characteristics of the autobiographical memory system. Finally, we used SAD as an example to illustrate specific applications of the SCIL model to clinical practice. Although the example used pertains to SAD, the model itself is conceptualized as being broadly applicable to a variety of clinical presentations, and we encourage scientists and clinicians to extend our predictions to other forms of psychopathology. Future well-designed brain-imaging and neurofeedback studies are needed to validate the SCIL model by linking the activation of proposed schema-congruent and -incongruent learning processes during treatment with activity in proposed neural regions (e.g., see K. D. Young et al., 2018).

Overall, the SCIL model highlights the importance and clinical utility of developing a deeper and more integrative understanding of autobiographical memory-related dysfunction in mental-health problems, such as SAD, that are characterized by maladaptive schemas that drive negative self-perception. Although we focused our clinical example on SAD, people who seek treatment for various types of problems, including other anxiety disorders, such as depression, eating disorders, and posttraumatic stress disorder, commonly experience symptoms of psychopathology that arise from overactive negative self-schemas. To this end, improving self-concept by engaging the autobiographical memory system through effective mental simulation should be considered a key transdiagnostic target for intervention science and practice.

## Transparency

*Action Editor:* Timothy J. Pleskac

*Editor:* Klaus Fiedler

*Author Contributions*

M. Moscovitch and S. Sheldon contributed equally. They are listed in alphabetical order by last name.

## Notes

1. We refer to prior work that functionally defines the vmPFC region involved in social inferences as the region lying below the 20-mm *z*-axis coordinate in the medial aspects of the PFC (Van Overwalle, 2009).

2. Although the bulk of the evidence is consistent with the view that gist and details are mediated, respectively, by the anterior and posterior hippocampus both in humans (Gilboa & Moscovitch, 2021; M. Moscovitch & Gilboa, in press) and rodents (Atucha, Ku, Lippert, & Sauvage, 2021; Jasnow et al., 2017), some studies have suggested the reverse is the case (Dandolo & Schwabe, 2018; Tompary & Davachi, 2017). Likewise, it is possible that simply engaging the gist-based processes in the anterior hippocampus leads to retrieval of a perceptually rich episodic memory or simulation even without engaging the posterior hippocampus. However, we think this is unlikely because research in human aging has shown a decline in episodic richness that is related to a decline in the posterior but not anterior hippocampus (Snytte et al., 2022). Likewise, loss of context-dependent but not gist memory in rodents is associated with damage or inactivation of posterior (dorsal in rodents) hippocampus despite preservation of anterior (ventral in rodents) hippocampus (Atucha et al., 2021; Jasnow et al., 2017). This is not the place to debate the relative merits of these proposals, but the interested reader is referred to Audrain et al. (2022), Audrain and McAndrews (2022), and M. Moscovitch and Gilboa (in press) for a more detailed discussion.

3. In Bayesian terms, clinicians might conceptualize the well-established negative schema at the start of treatment as one that has very strong priors based on the person’s past experiences and that new inconsistent evidence (posteriors) is likely to have a relatively weak impact. In contrast, the fragile positive schema could be conceptualized at the start of treatment as one with very weak priors based on the person’s past experiences and that new supporting evidence (posteriors) has the potential for a relatively strong impact, if optimally encoded.

4. In this section, we do not aim to undertake a comprehensive review of the neurocognitive literature pertaining to SAD and its treatment. Indeed, such reviews have been published elsewhere (e.g., Cremers & Roelofs, 2016) and would be well beyond the scope of the present article, particularly given the complexity of findings across numerous studies that have shown differential effects depending on the nature of the treatment context and the experimental tasks investigated (e.g., Ziv et al., 2013).

5. Treatment of SAD may also be conceptualized within a Pavlovian fear-conditioning framework aligned with Craske et al.’s (2008, 2014) inhibitory-learning model. For example, the conditioned stimulus (CS) may be conceptualized as the social context, the unconditioned stimulus (US) may be conceptualized as negative evaluation, and the conditioned responses (CR) may be conceptualized as emotional distress and behavioral avoidance. In Pavlovian terms, the CR is elicited consistently after the CS becomes a reliable predictor of the US. Within this framework, the primary goal of treatment for SAD can be conceptualized as weakening patients’ association between CS and US. Following our model, weakening this CS–US association can be achieved by guiding patients to learn and mentally simulate new contingencies that are inconsistent with the conclusion that social situations (CS) inevitably lead people to judge them as being socially undesirable (US), which should ultimately reduce the CRs that are elicited in the presence of social scrutiny while strengthening new CS–US associations that link relevant social contexts with positive self-perception and adaptive emotional and behavioral outcomes.
